# Proposal of an automated tumor‐stromal ratio assessment algorithm and a nomogram for prognosis in early‐stage invasive breast cancer

**DOI:** 10.1002/cam4.4928

**Published:** 2022-06-11

**Authors:** Qian Xu, Yuan‐Yuan Chen, Ying‐Hao Luo, Jin‐Sen Zheng, Zai‐Huan Lin, Bin Xiong, Lin‐Wei Wang

**Affiliations:** ^1^ Department of Radiation and Medical Oncology Zhongnan Hospital of Wuhan University Wuhan China; ^2^ Department of Gastrointestinal Surgery Zhongnan Hospital of Wuhan University Wuhan China; ^3^ Hubei Key Laboratory of Tumor Biological Behaviors Hubei Cancer Clinical Study Center Wuhan China

**Keywords:** breast cancer, image analysis algorithm, qupath, tumor stromal ratio

## Abstract

**Background:**

The tumor‐stromal ratio (TSR) has been verified to be a prognostic factor in many solid tumors. In most studies, it was manually assessed on routinely stained H&E slides. This study aimed to assess the TSR using image analysis algorithms developed by the Qupath software, and integrate the TSR into a nomogram for prediction of the survival in invasive breast cancer (BC) patients.

**Methods:**

A modified TSR assessment algorithm based on the recognition of tumor and stroma tissues was developed using the Qupath software. The TSR of 234 invasive BC specimens in H&E‐stained tissue microarrays (TMAs) were assessed with the algorithm and categorized as stroma‐low or stroma‐high. The consistency of TSR estimation between Qupath prediction and pathologist annotation was analyzed. Univariable and multivariable analyses were applied to select potential risk factors and a nomogram for predicting survival in invasive BC patients was constructed and validated. An extra TMA containing 110 specimens was obtained to validate the conclusion as an independent cohort.

**Results:**

In the discovery cohort, stroma‐low and stroma‐high were identified in 43.6% and 56.4% cases, respectively. Good concordance was observed between the pathologist annotated and Qupath predicted TSR. The Kaplan–Meier curve showed that stroma‐high patients were associated with worse 5‐DFS compared to stroma‐low patients (*p* = 0.007). Multivariable analysis identified age, T stage, N status, histological grade, ER status, HER‐2 gene, and TSR as potential risk predictors, which were included in the nomogram. The nomogram was well calibrated and showed a favorable predictive value for the recurrence of BC. Kaplan–Meier curves showed that the nomogram had a better risk stratification capability than the TNM staging system. In the external validation of the nomogram, the results were further validated.

**Conclusions:**

Based on H&E‐stained TMAs, this study successfully developed image analysis algorithms for TSR assessment and constructed a nomogram for predicting survival in invasive BC.

## Graphical abstract

This study aimed to assess the TSR using image analysis algorithms developed by the Qupath software, and include the TSR into a nomogram for prediction of the survival in invasive breast cancer (BC) patients.

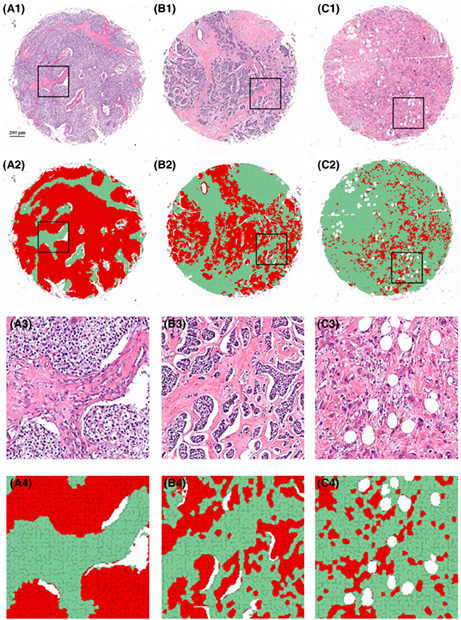

## 1 INTRODUCTION

Recent studies indicate that breast cancer (BC) is the most commonly diagnosed cancer in the world, with an estimated 2,261,419 new cases and 684,996 new deaths in 2020.^1^ Despite significant advances in diagnostic tools and treatment techniques, the incidence, prevalence, and mortality of patients with BC remain high in the past decades.^2^, ^3^ It is necessary to identify novel prognostic factors to optimize risk stratification and guide treatment decisions.

Growing interest has emerged in the bidirectional interactions between tumor and stroma.^4^ On the one hand, malignant cells are actively involved in the activation of non‐malignant cells and the remodeling of the extracellular matrix. On the other hand, these components in the stroma promote the proliferation, invasion, and metastasis of malignant cells through secreting various growth factors, chemokines, and cytokines.^5^, ^6^, ^7^ The bidirectional interactions between tumor and stroma play a vital role in tumor development, immunosuppression, angiogenesis, and drug tolerance.^8^, ^9^ As a result, the tumor‐stromal ratio (TSR), a parameter representing the relative amounts of tumor and surrounding stroma, was introduced to the field of cancer research. Previous studies have demonstrated that the TSR was of prognostic significance in cases of colon carcinoma,^10^ rectal adenocarcinoma,^11^ hepatocellular carcinoma,^12^ non‐small cell lung cancer,^13^ gallbladder cancer,^14^ and BC.^15^

Currently, the TSR is assessed on routinely stained H&E slides with two methods, visual assessment (‘eyeballing’)^16^, ^17^ and systematic point counting.^18^, ^19^ Visual assessment of the TSR is performed by independent investigators, using a previously determined cut‐off value of 50% to delineate a high or low stroma content tumor. The systematic point counting method employs the Randomspot tool to superimpose a group of 300 random points on the selected area in whole tumor slides. Each point is pathologically categorized as ‘tumor,’ ‘stroma,’ or ‘non‐informative’. The ultimate TSR is determined as the number of points, categorized as ‘stroma’ divided by the total number of points. Although good inter‐observer agreement was found in these studies, visual assessment of the TSR may still suffer from reproducibility issues.^15^, ^20^ Furthermore, both methods require vast workflow of investigators to manually categorize the histopathology when assessing large numbers of histological slides.

To overcome the disadvantages of visual assessment, this study employed an open‐source software platform (the Qupath software)^21^ to develop a modified TSR assessment algorithm based on the recognition of tumor and stroma tissues. The digital image analysis algorithm could provide a solution to the problems of standardization and observer variation caused by subjectivity. The high throughput analysis of tissue cores in tissue microarrays (TMAs) could also largely facilitate the workflow of investigators. The aim of the study was to assess the TSR using image analysis algorithms developed by the Qupath software and integrate the TSR into a nomogram for predicting the survival of invasive BC patients.

## 2 METHODS

### 2.1 Patient cohorts and TMAs preparation

A comprehensive database of BC has been established at our center, which was the data source of several clinical and translational studies.^22^, ^23^ From the database, 240 invasive BC patients were selected who were primarily treated with surgery after diagnosis from January 2002 to December 2006. Major clinical and pathological factors were available, including age, tumor size, lymph node status, histological type, ER status, HER2 status, and TNM staging. All specimens were histologically graded according to the WHO histological grading.^24^ Disease‐free survival (DFS) was used as the primary endpoint and defined as the time period between date of surgery and date when locoregional recurrence or distant metastasis occurred.

TMAs were prepared with standard procedures collaborating with Shanghai Outdo Biotech (Shanghai, China), as previously described.^25^ All specimens were reviewed on diagnostic H&E slides and the most invasive tumor areas containing tumor tissue and surrounding stroma tissue were selected. Corresponding areas were marked on the original FFPE block for cutting. Two cores were taken from marked areas of each paraffin block using punch cores. The cores were then deposited into recipient paraffin blocks with 70 cylinders. Seven TMAs blocks containing 480 cores were constructed and cut into 4 μm sections, with one slide every 50 retained for H&E staining and quality control. The study protocol was approved by the Institutional Ethics Committee of Zhongnan Hospital of Wuhan University (Scientific Ethical Approval No. 2017057). In addition, an extra TMA containing paraffin‐embedded specimens of 110 invasive BC patients from January 2005 to July 2009 was obtained from Outdo Biotech (Shanghai, China), which were used to validate the conclusion as an independent cohort.

### 2.2 Digital scanning, algorithm training and assessment of the TSR

**FIGURE 1:**
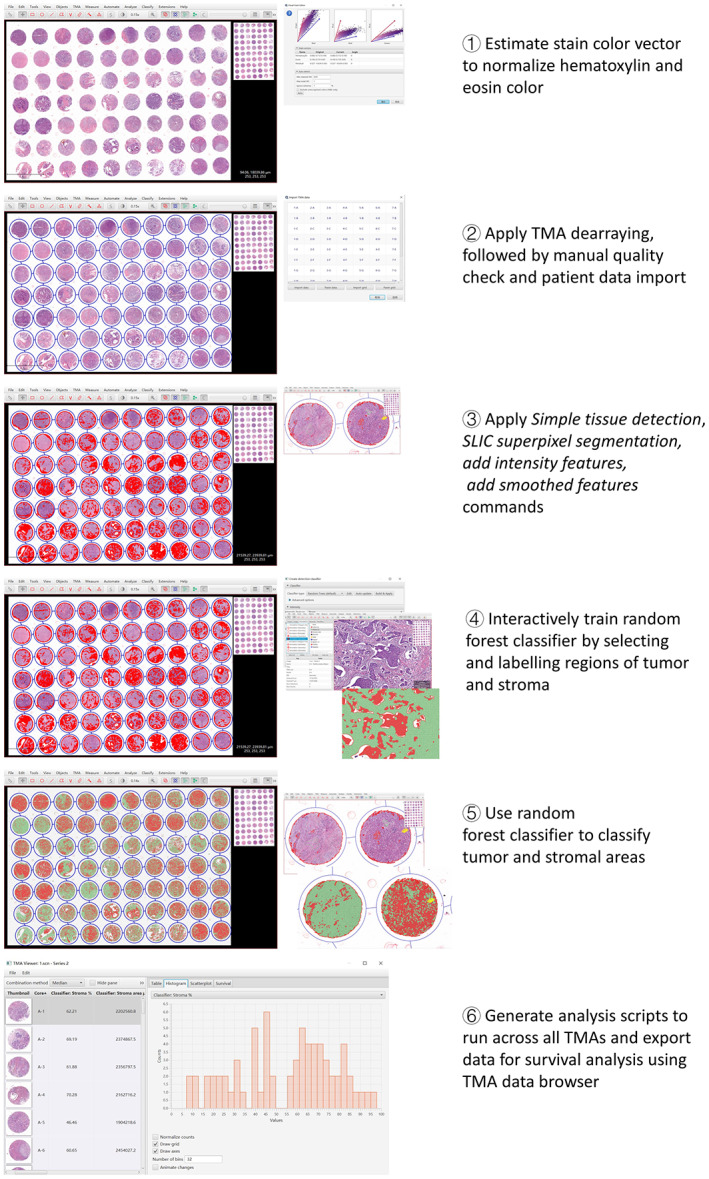
Workflow of algorithm training and analysis with the Qupath software on TMAs

All TMAs were stained with H&E staining technique and scanned at ×200 total magnification (0.271 μm/pixel) using the Aperio VERSA automated slide scanner (Leica Biosystems Imaging). Each TMA was scanned into a digital image with the image type of “.scn files”, which represented the ScanScope Virtual Slide files. This image file is fully compatible with the Aperio software portfolio, enabling remote review of slides and sophisticated image analysis. Qupath, an open‐source digital image analysis software platform (version 0.2.3, https://qupath.github.io/), was employed to develop an automated TSR assessment algorithm. Workflow of algorithm training with the Qupath software and following TMAs analysis is shown in Figure 1, which comprises the following steps, ① Estimated stain vector was first defined to normalize hematoxylin and eosin color after uploading digital images of TMAs (.scn files); ② Module “*TMA derrayer*” was used to appropriately orientate cores of TMAs. Then, manual quality check was performed to exclude a few cores with any folded, blurred, or defective morphology; ③ Command “*Simple tissue detection*” was applied to accurately detect the tissue of each core. Commands “*SLIC superpixel segmentation*”, “*Add intensity features*” were applied to generate ‘superpixels’ and calculate corresponding features (Table S1). The specific settings used in these commands were previously described.^21^ Command “*Add smoothed feathers*” was applied to add smoothed object features at 50 μm radius to improve the accuracy of classification; ④ Representative training areas, which comprise tumor tissue, stroma tissue, and “other” (whitespace, necrosis, mucin, inflammation, etc.), were annotated accordingly by an experienced investigator (LW Wang) using QuPath's annotation tools; ⑤ Algorithm training of the random forest classifier was performed to achieve the optimal classification of tumor tissue and surrounding stromal tissue; ⑥ The trained algorithm was then applied to all TMAs sequentially. Data output using the TMA data browser provided the area of tumor tissue and stroma tissue (μm^2^) in each core, respectively. The TSR was calculated as TSR = Area of stroma/(Area of tumor + Area of stroma) × 100%. The higher stromal ratio from two cores of each specimen was considered as the ultimate TSR.

### 2.3 The consistency analysis

From 480 cores in seven TMAs, 30% (*n* = 145) image fields (×100 magnification) were randomly selected. The trained algorithm (trained by Q. Xu and L. W. Wang) was applied to 145 image fields to assess the TSR. The result was recorded as TSR‐algorithm. On the other hand, another investigator (Y. Y. Chen) manually outlined the tumor area (in black) and stroma area (in white) on 145 image fields using the ImageJ software (version 1.51). The percentage of stroma was then calculated, and the result was recorded as TSR‐investigator. TSR consistency analysis was performed by assessing the concordance between TSR‐algorithm and TSR‐investigator. The correlation coefficient (Pearson r) and intra‐class correlation coefficient (ICC) were calculated. The Bland–Altman plot was used to assess the between‐method agreement.

### 2.4 Statistical analysis

The association between clinicopathological factors and TSR was evaluated using Pearson *χ* ^2^ test or Fisher's exact test. Kaplan–Meier curves and the log‐rank test were applied for survival analysis. Unadjusted HRs (hazard ratios) with 95% CIs (confidence intervals) of TSR associated with 5 years disease‐free survival (5‐DFS) were calculated using the Cox regression model. Univariable and multivariable analyses of risk factors for 5‐DFS were performed. Potential risk factors were selected in the univariable analysis. A statistical significance level of 0.10 was used to select risk factors into the best‐fit model in the multivariable analysis. Subsequently, based on the results of multivariable analysis, a nomogram was depicted to generate probability of 5‐DFS. Concordance index (C‐index), calibration plot and decision curve analysis (DCA) were used to evaluate the discrimination and calibration of the nomogram. The ROC curve was plotted to compare the predictive value of the nomogram and the TNM staging system. In addition, Kaplan–Meier curves were used to estimate the probability of DFS between risk subgroups of the nomogram and the TNM staging system. Statistical analyses were performed using IBM SPSS version 23.0 (SPSS Inc.) and R 3.6.3 software (https://cran.r‐project.org/) with the following packages: survival, Hmisc, lattice, rms, ggplot2, ggDCA.

## 3 RESULTS

### 3.1 The consistency analysis

**FIGURE 2:**
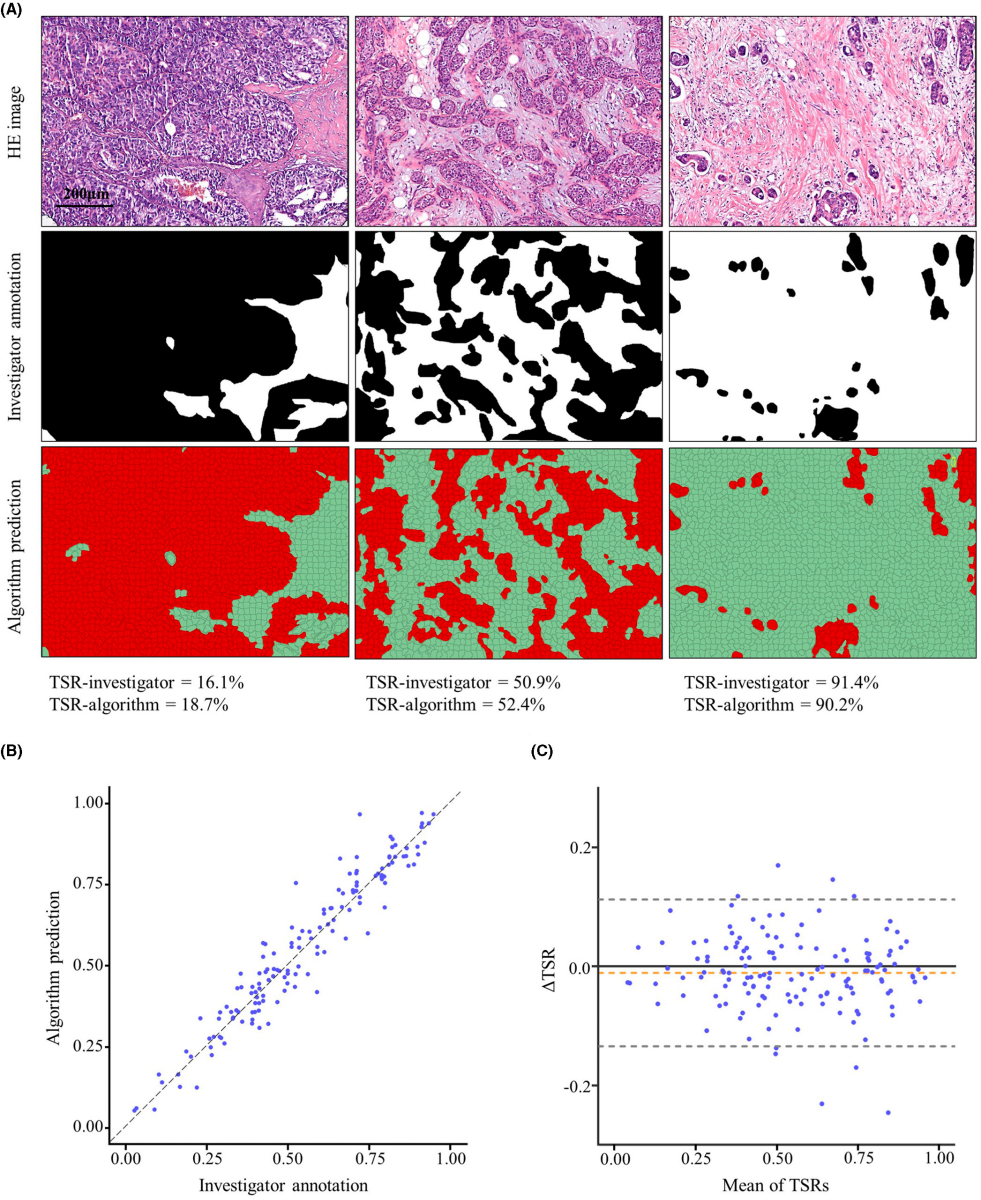
TSR consistency analysis. (A) Representative image fields of investigator annotation (tumor in black, stroma in white) and algorithm prediction (tumor in red, stroma in green). (B) Concordance between TSR‐algorithm and TSR‐investigator. (C) Bland–Altman plot for assessing between‐method agreement. The solid horizontal line is the mean, the dashed line is zero, and the shaded regions are 95% CIs

For TSR consistency analysis, representative image fields of investigator annotation and algorithm prediction were shown in Figure 2A. There was a high agreement (ICC = 0.960, 95% CI 0.945–0.971) between the TSR predicted by the algorithm and the investigator. Good concordance was also observed between TSR‐algorithm and TSR‐investigator (Pearson *r* = 0.960, *p* < 0.01) (Figure 2B). In addition, Bland–Altman plot showed good agreement between two methods (Figure 2C). The mean difference of TSRs (algorithm vs. investigator) was −0.01 (95% CI −0.13–0.11).

### 3.2 H&E images and corresponding algorithm prediction results

**FIGURE 3:**
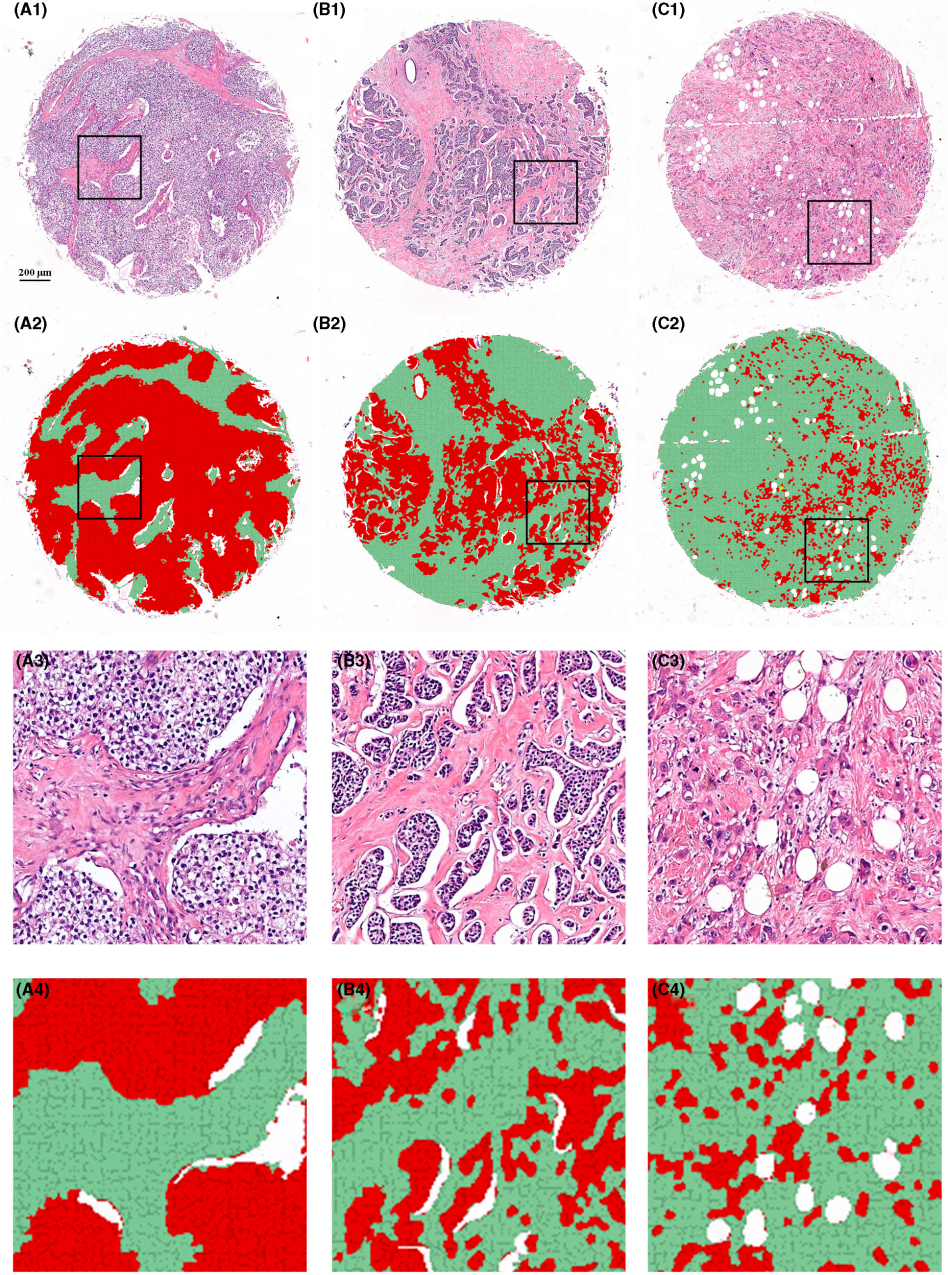
Representative H&E images (A1, B1, and C1) and corresponding algorithm prediction results (A2, B2, and C2) in TMAs. The TSR of the three cores (A1, B1, and C1) were 14.1%, 55.6%, and 86.5%, respectively. At high power magnification (400 × 400 μm) (A4, B4, and C4), tumor tissue and surrounding stroma tissue were naturally segmented in separate colors (tumor in red, stroma in green, none‐cell areas in white). H&E, hematoxylin–eosin; TSR, tumor‐stromal ratio

Representative H&E images and corresponding algorithm prediction results in TMAs were shown in Figure 3. Using the trained algorithm, tumor tissue (in red), stroma tissue (in green), and “other” (in white) were segmented (A2, B2, and C2). Panels A1 and A2 showed a core with low percentage of tumor‐associated stroma, estimated 14.1% stroma ratio. Panels B1 and B2 showed a core with middle percentage of tumor‐associated stroma, estimated 55.6% stroma ratio. Panels C1 and C2 showed a core with high percentage of tumor‐associated stroma, estimated 86.5% stroma ratio. At high power magnification (400 × 400 μm) (A4, B4, and C4), we observed that tumor tissue and surrounding stroma tissue were naturally segmented in separate colors.

### 3.3 Assessment of tumor‐stromal ratio and its correlation with major clinicopathological factors

The trained algorithm was applied to assess the TSR in 234 specimens, with 6 specimens excluded due to their cores with folded, blurred, or defective morphology. The median TSR was 57.0% and the mean of TSR was 57.9%. A cut‐off value of 50% was set, which was in line with previous studies.^26^ Out of 234 specimens, 43.6% (*n* = 102) were determined as stroma‐low and 56.4% (*n* = 132) as stroma‐high. The median age at primary surgery was 48 (29–78) years. Major clinicopathological factors stratified by the TSR categories were shown in Table 1. High stroma content was correlated with HER2 gene status (*p* = 0.029), but no significant difference regarding age, tumor size, lymph node status, and histological type was observed (Table 1). In addition, no correlation was found between the TSR and the molecular or histological types (Figure S1).

**TABLE 1:** The relationship between the TSR and major clinicopathological factors

Characteristics	Total, *n* (%)	Stroma low, *n* (%)	Stroma high, *n* (%)	*p* value
Age (years)				0.862
≤50	146 (62.4)	63 (61.8)	83 (62.9)	
>50	88 (37.6)	39 (38.2)	49 (37.1)	
Menopausal status				0.228
Premenopausal	132 (56.4)	53 (52.0)	79 (59.8)	
Postmenopausal	102 (43.6)	49 (48.0)	53 (40.2)	
Histological type				0.898
No special type	189 (80.8)	82 (80.4)	107 (81.1)	
Others	45 (19.2)	20 (19.6)	25 (18.9)	
T stage (cm)				0.379
T1 (*T* ≤ 2)	35 (15.0)	18 (17.6)	17 (12.9)	
T2 (2 < *T* ≤ 5)	157 (67.1)	69 (67.6)	88 (66.7)	
T3 (*T* > 5)	42 (17.9)	15 (14.7)	27 (20.4)	
N status				0.117
N negative	108 (46.2)	53 (52.0)	55 (41.7)	
N positive	126 (53.8)	49 (48.0)	77 (58.3)	
Histological grade				0.283
I	39 (16.7)	20 (19.6)	19 (14.4)	
II	137 (58.5)	59 (57.9)	78 (59.1)	
III	58 (24.8)	23 (22.5)	35 (26.5)	
ER status				0.410
Negative	131 (56.0)	54 (52.9)	77 (58.3)	
Positive	103 (44.0)	48 (47.1)	55 (41.7)	
PR status				0.463
Negative	129 (55.1)	59 (57.8)	70 (53.0)	
Positive	105 (44.9)	43 (42.2)	62 (47.0)	
HER2 gene				0.029
Non‐amplification	184 (78.6)	87 (85.3)	97 (73.5)	
Amplification	50 (21.4)	15 (14.7)	35 (26.5)	

Abbreviations: BC, breast cancer; ER, estrogen receptor; HER2, human epidermal growth factor receptor‐2; N, node; T, tumor; TSR, tumor‐stromal ratio.

### 3.4 Survival analysis and subgroup analysis in relation to the TSR

**FIGURE 4:**
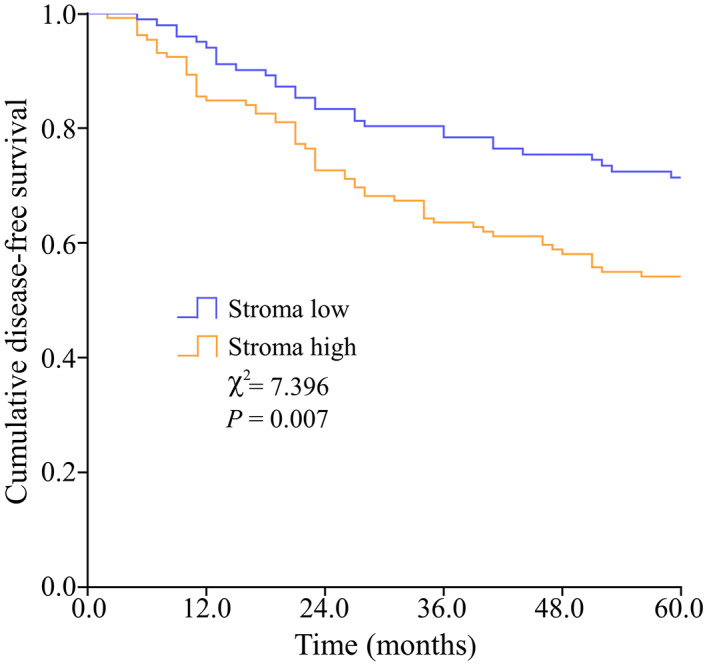
The Kaplan–Meier curve for stroma‐low and stroma‐high groups. Patients with high stroma had worse disease‐free survival compared with patients with low stroma (*χ* ^2^ = 7.396, *p* = 0.007)

For the included cohort (*n* = 234), the 5‐DFS rate was 62.0%. The Kaplan–Meier curve for stroma‐low and stroma‐high groups was shown in Figure 4. Patients of high stroma had worse disease‐free survival compared with patients of low stroma (*χ* ^2^ = 7.396, *p* = 0.007), with the 5‐DFS rate of 54.5 vs. 71.6%, respectively.

**FIGURE 5:**
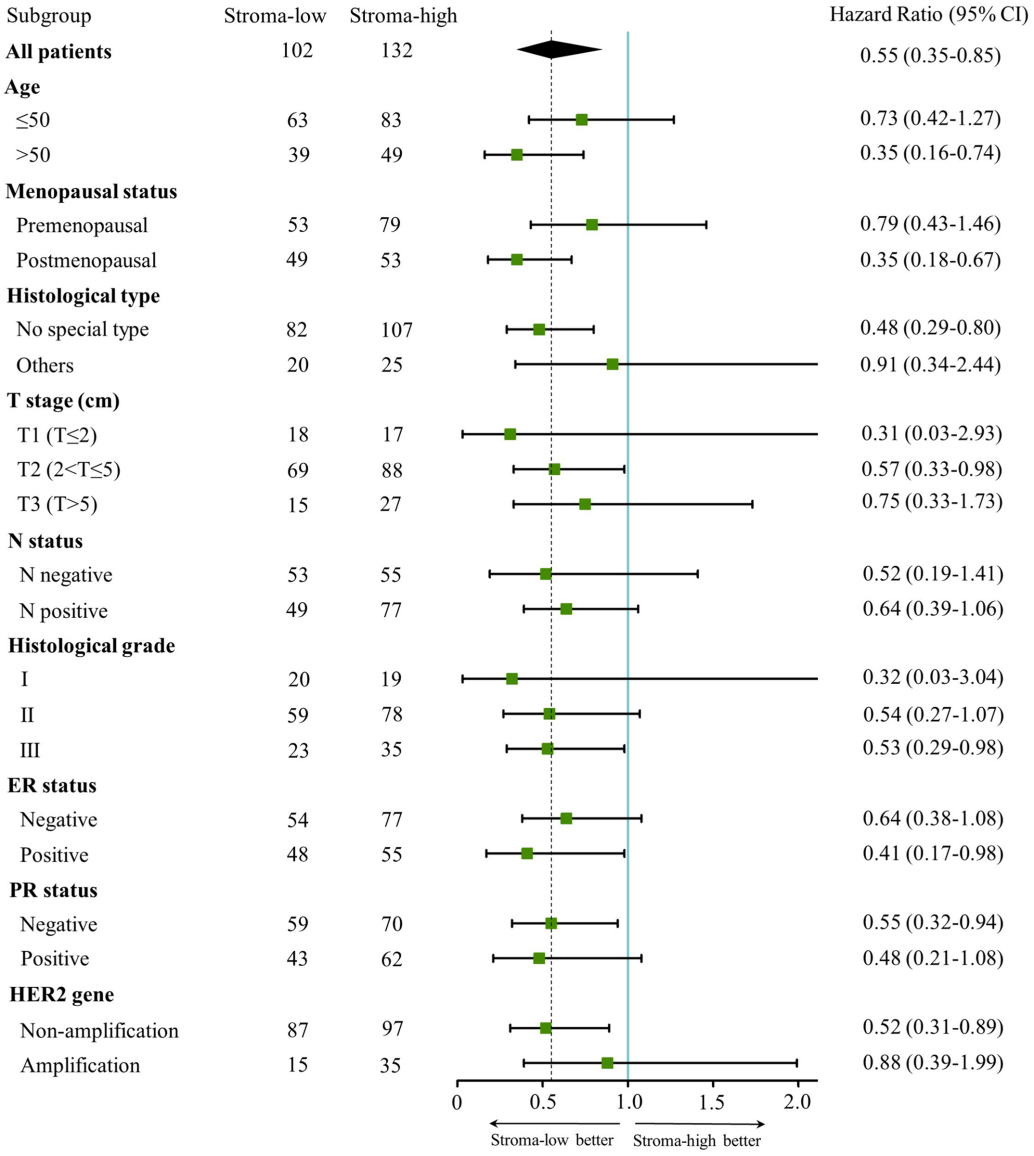
Forest plot of subgroup analysis in stroma‐low and stroma‐high groups with 5‐DFS. The dashed line is 0.55 representing the hazard ratio in all patients

Subgroup analysis of the TSR for 5‐DFS was shown in Figure 5. High TSR was consistently correlated with worse 5‐DFS in age > 50 (HR 0.35; 95% CI 0.16–0.74; *p* = 0.006), postmenopausal (HR 0.35; 95% CI 0.18–0.67; *p* = 0.002), ductal carcinoma (HR 0.48; 95% CI 0.29–0.80; *p* = 0.004), T2 (HR 0.57; 95% CI 0.33–0.98; *p* = 0.043), histological Grade III (HR 0.53; 95% CI 0.29–0.98; *p* = 0.043), ER positive (HR 0.41; 95% CI 0.17–0.98; *p* = 0.045), PR negative (HR 0.55; 95% CI 0.32–0.94; *p* = 0.028), and HER2 gene non‐amplification subgroups (HR 0.52; 95% CI 0.31–0.89; *p* = 0.016). Furthermore, a non‐significant correlation was found between high stroma and worse 5‐DFS (*p* > 0.05) in age ≤ 50, premenopausal, T3, N status, histological Grade II, ER negative, PR positive, and HER2 gene amplification subgroups.

### 3.5 Univariable and multivariable analysis of the TSR and other factors

Potential risk factors were selected in the univariable analysis. The associations between 5‐DFS and potential risk factors were further examined by multivariable analysis. Factors including age (*p* = 0.077), T stage (*p* = 0.030), N status (*p* < 0.001), histological grade (*p* < 0.001), ER status (*p* = 0.066), HER‐2 gene (*p* = 0.024) and TSR (*p* = 0.049) were separately incorporated to develop the nomogram (Table 2).

**TABLE 2:** Multivariable analysis for 5‐DFS and the C‐index of each factor

Parameters	Multivariable analysis			C‐index (95% CI)
	HR	95% CI	*p* value	
Age (years)				0.521 (0.469–0.572)
≤50	1.000			
>50	1.482	0.958–2.294	0.077	
Histological type				
No special type	1.000			
Others	1.112	0.642–1.927	0.705	
T stage (cm)				0.624 (0.575–0.674)
T1 (*T* ≤ 2)	1.000			
T2 (2 < *T* ≤ 5)	3.227	1.165–8.939	0.024	
T3 (*T* > 5)	4.198	1.444–12.204	0.008	
*N* status				0.681 (0.637–0.724)
Negative	1.000			
Positive	3.905	2.248–6.781	< 0.001	
Histological grade				0.714 (0.669–0.759)
I	1.000			
II	1.578	0.554–4.499	0.393	
III	5.391	1.836–15.830	0.002	
ER status				0.607 (0.559–0.655)
Negative	1.000			
Positive	0.628	0.383–1.031	0.066	
HER2 gene				0.586 (0.538–0.634)
Non‐amplification	1.000			
Amplification	1.773	1.077–2.921	0.024	
TSR				0.574 (0.523–0.624)
Low TSR	1.000			
High TSR	1.603	1.001–2567	0.049	

Abbreviations: ER, estrogen receptor; HER2, human epidermal growth factor receptor‐2; N, node; T, tumor; TSR, tumor‐stromal ratio.

### 3.6 Development and validation of nomogram for predicting the probability of 5‐DFS

**FIGURE 6:**
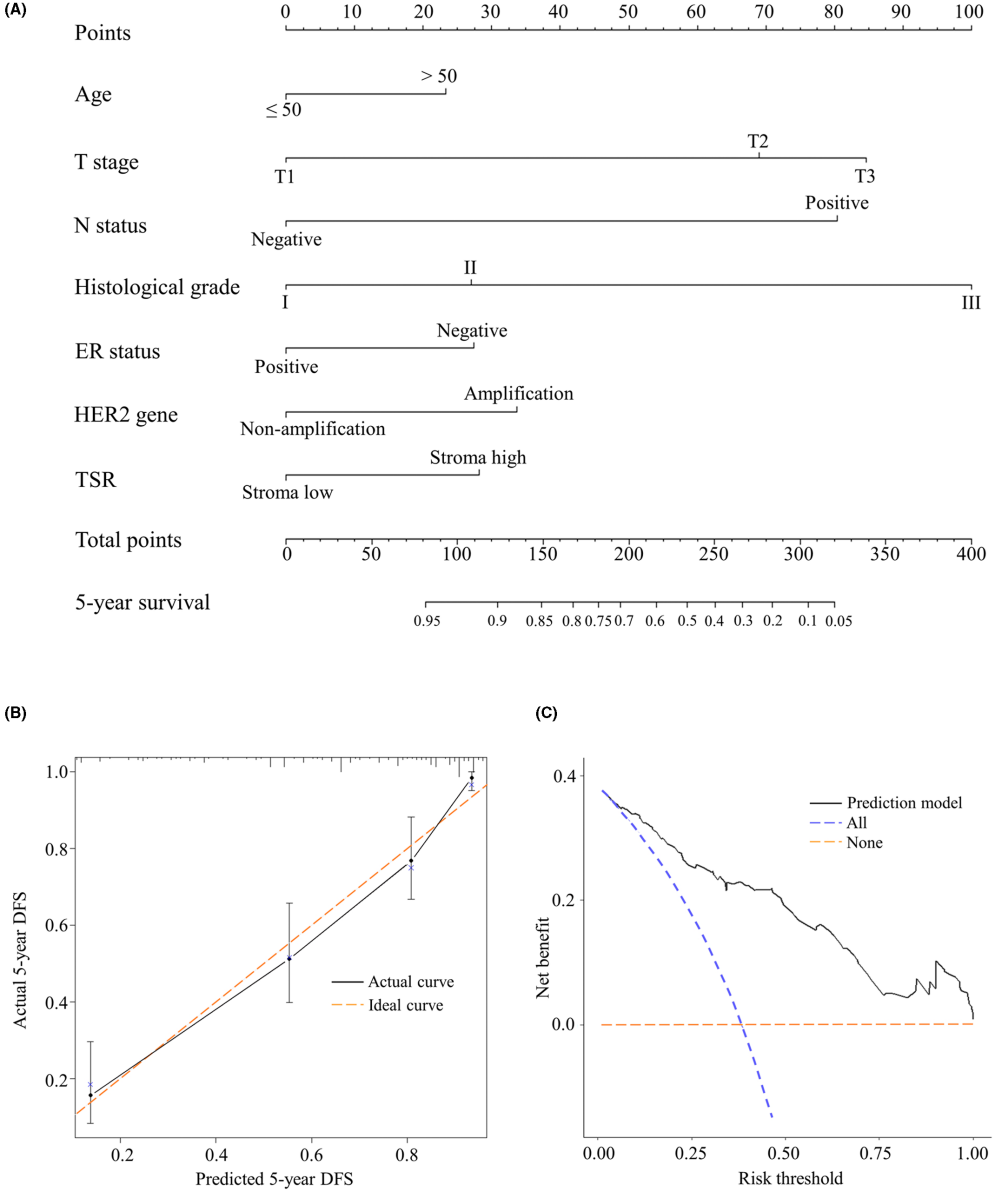
Development and validation of nomogram. (A) Nomogram predicting the probability of 5‐DFS in invasive BC patients. (B) Calibration plot of the nomogram. (C) Decision curve analysis of the nomogram

Based on the results of multivariable analysis, a nomogram was depicted to generate the probability of 5‐DFS (Figure 6A). The score of each factor was calculated drawing an upward vertical line from the factor to the “points” line. According to the sum points of each factor, a downward vertical line was drawn to obtain the probability of 5‐DFS. The detailed score of each factor was as follows, age (age ≤ 50: 0.0, age > 50: 23.2), T stage (T1: 0.0, T2: 69.0, T3: 84.7), N status (negative: 0.0. positive: 80.4), histological grade (I: 0.0, II: 27.0, III: 100.0), ER status (positive: 0.0, negative: 27.4), HER‐2 gene (non‐amplification: 0.0, amplification: 33.6), and TSR (stroma‐low: 0.0, stroma‐high: 28.1). The nomogram demonstrated a good discriminative ability with the C‐index of 0.821 (95% CI: 0.781–0.861), which was higher than the C‐index of any single risk factor (Table 2). The calibration plot exhibited favorable consistencies between the observed outcomes and nomogram predictions about the probability of 5‐DFS (Figure 6B). The DCA exhibited preferable net benefits of the nomogram, along with the threshold probabilities (Figure 6C).

### 3.7 Comparison of the nomogram and the TNM staging system

**FIGURE 7:**
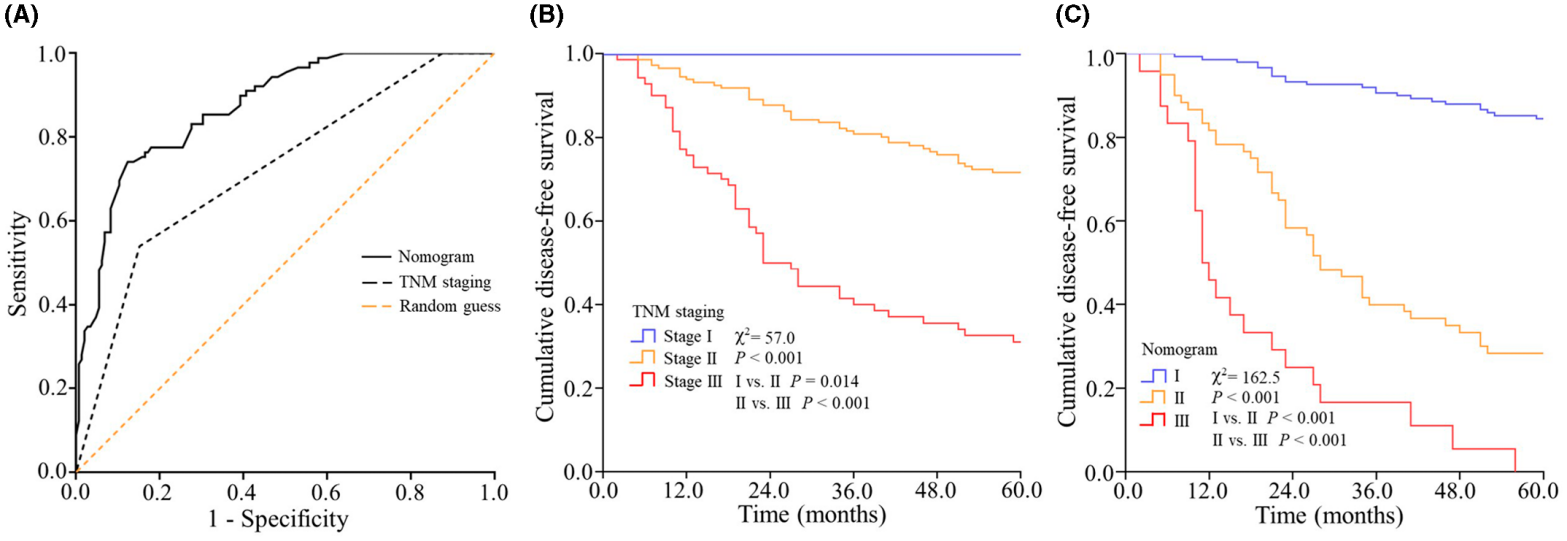
Comparison of in the nomogram and the TNM staging system using the ROC analysis and Kaplan–Meier curves. The ROC curve of the nomogram and the TNM staging system (A). The Kaplan–Meier survival curves estimating the probability of DFS between risk subgroups of the TNM staging system (B) and the nomogram (C)

Predictive value of the TNM staging system and the nomogram was studied by the ROC analysis. The AUC of the nomogram (0.873; 95% CI: 0.828–0.917) was larger than that of the TNM staging system (0.722; 95% CI: 0.655–0.790), which indicated a better predictive value of the nomogram (*p* < 0.001) (Figure 7A). Furthermore, for all patients, the sum points of each factor in the nomogram were calculated. Optimal cut‐off value for the sum points was identified (227.7, 304.4) using the X‐tile software and patients with sum points ≤ 227.7, 227.7 < sum points ≤ 304.4, and sum points > 304.4 were categorized into I (*n* = 150), II (*n* = 60), and III (*n* = 24) subgroups in the nomogram. Kaplan–Meier curves were then used to estimate the probability of DFS between risk subgroups of two prediction models (the TNM staging system and the nomogram), which indicated a better risk stratification of the nomogram (*χ* ^2^ = 162.5, *p* < 0.001 for I vs. II and II vs. III) (Figure 7B) than the TNM staging system (*χ* ^2^ = 57.0, *p* = 0.014 for Stage I vs. Stage II, *p* < 0.001 for stage II vs. stage III) (Figure 7C).

### 3.8 Validation of the predictive value of TSR in an independent cohort

**FIGURE 8:**
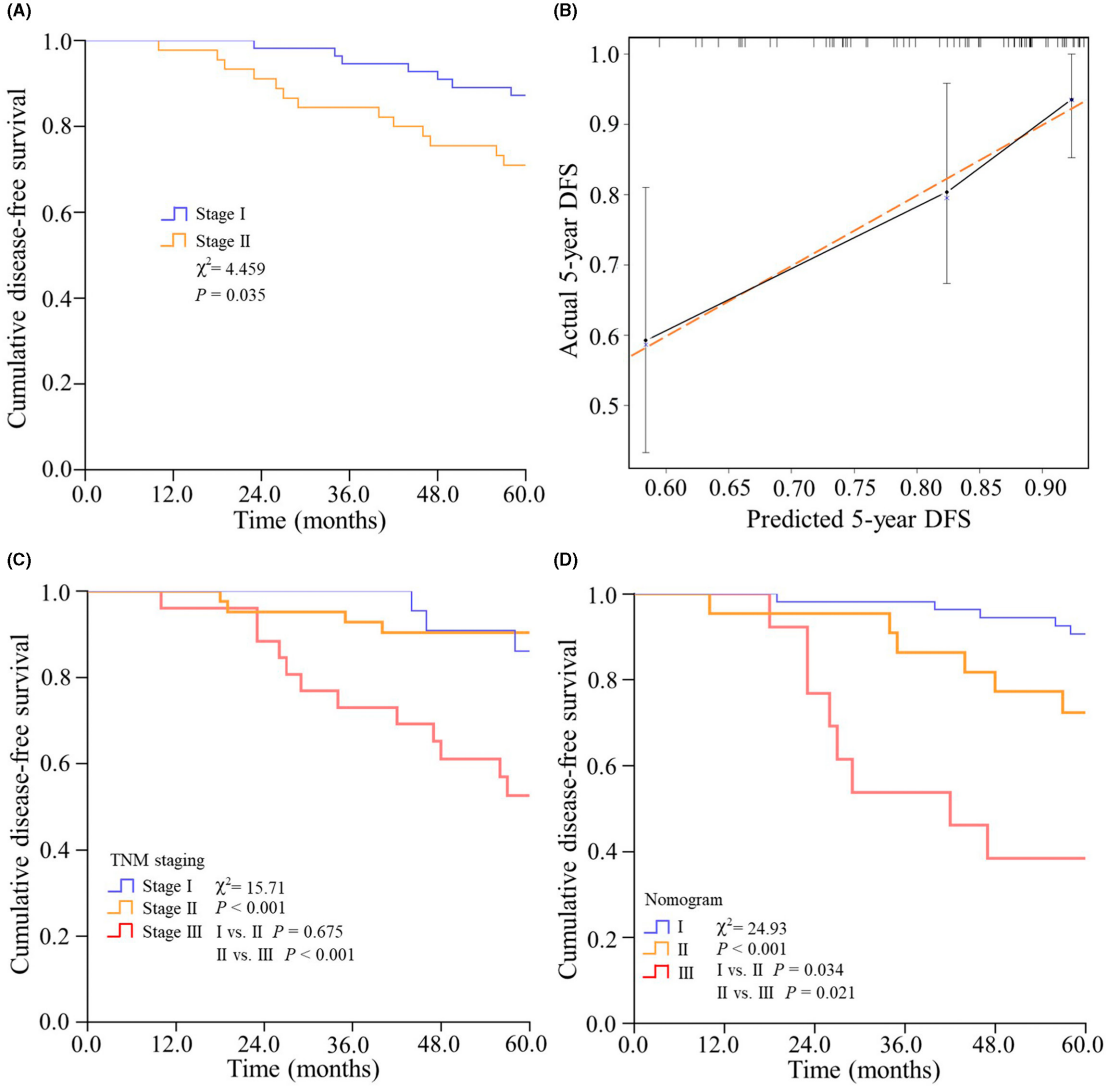
Validation of the predictive value of TSR in an independent cohort. The Kaplan–Meier curve for stroma‐low and stroma‐high groups in the validation cohort (A). Calibration plot of the nomogram in the validation cohort (B). The Kaplan–Meier survival curves estimating the probability of DFS between risk subgroups of the TNM staging system (C) and the nomogram (D) in the validation cohort

TMAs containing 110 paraffin‐embedded invasive BC specimens were obtained as an independent cohort to validate the predictive value of the TSR. Among 110 specimens, 9 specimens were excluded due to their cores with folded, blurred, or defective morphology. Major clinicopathological factors stratified by the TSR categories were shown in Table S2. As expected, patients of high stroma had worse DFS compared with patients of low stroma (*χ* ^2^ = 4.459, *p* = 0.035) (Figure 8A). In the validation of the nomogram, the calibration plot exhibited favorable consistencies between the observed outcomes and nomogram predictions (Figure 8B). The ROC analysis demonstrated a larger AUC of the nomogram (0.759, 95% CI: 0.637–0.881) than the TNM staging system (0.703, 95% CI: 0.558–0.849). Furthermore, Kaplan–Meier curves indicated a better risk stratification of the nomogram (*χ* ^2^ = 24.93, *p* = 0.034 for I vs. II, Figure 8D) than the TNM staging system (*χ* ^2^ = 15.71, *p* = 0.675 for stage I vs. stage II, Figure 8C).

## 4 DISCUSSION

The bidirectional interactions between tumor and stroma are actively involved in the tumor proliferation, invasion, and metastasis. In previous studies, the TSR has been acknowledged to have prognostic significance in BC,^17^, ^27^, ^28^ indicating an association between high stroma content and poor prognosis. Despite this evidence, no implementation of the TSR was found in routine pathology reporting. This might be attributed to the variation in methodology and the lack of a standardized procedure for TSR assessment.^20^

For the methods applied to TSR assessment, published studies have proposed visual assessment (“eyeballing”) and systematic point counting. Good inter‐observer agreement was observed with Cohen's kappa value varying from 0.68 to 0.85.^15^ However, visual assessment of the TSR might still suffer from reproducibility issues. In addition, when assessing the large number of histological slides, both methods required vast workflow of investigators to manually categorize the histopathology. In our previous studies, the prognostic value of the TSR was evaluated in invasive BC on slides with immunohistochemistry (IHC) staining of cytokeratin,^26^ and a computerized method was further developed to calculate the TSR.^25^ Herein, to achieve an automated methodology for TSR assessment on H&E‐stained slides, this study employed an open‐source software platform (the Qupath software) to develop a modified TSR assessment algorithm.

The Qupath software contained built‐in algorithms for common tasks, including cell and tissue detection. It allowed researchers to perform interactive machine learning using powerful annotation and visualization tools for object and pixel classification.^29^ In addition, QuPath used analysis scripts to represent sequences of steps that have been applied to an image. This included not only the commands that were run, but also the parameters that were used. Analysis scripts therefore, provided a way to record and standardize how an image was analyzed and to see how the results were generated. Then the same steps can be applied to other images.^30^ In this study, by selecting and annotating regions of interest (tumor, stroma, and “other”), algorithm training of the random forest classifier was performed to achieve an optimal classification. After training the classifier with one or more slides, the resultant scripts could be saved and then applied to new images without further training required.

With advances in digital image analysis, several automatic methods were applied to evaluate parameters related to the microenvironment, like lymphocytes, macrophages, tumor budding,^31^, ^32^ and the TSR. Ewan et al.^33^ described the use of a pixel classifier to automatically assess the stroma ratio in luminal and triple‐negative breast cancer, which supported the finding of high stroma content as an adverse prognostic factor. Ke et al.^34^ trained a convolutional neural network (CNN) model to quantify the TSR on H&E‐stained whole‐slide images of colorectal cancer and achieved a nine‐class tissue classification performance. A similar approach based on CNN model was developed to segment relevant tissue classifications (tumor, stroma, necrosis, other tissue) in rectal cancer and indicated improved predictive power over expert human visual assessment.^20^ However, the CNN model requires massive deep learning processes for model training and large sample of data set for subsequent validation.^35^, ^36^ The QuPath software, in contrast, provides a ready‐made, user‐friendly platform for algorithm training and modification. Therefore, this study employed the Qupath software to develop a modified algorithm based on the recognition of tumor and stroma tissues. It aimed to assess the TSR and determine its prognostic significance in invasive BC.

Using the TSR assessment algorithm, the tumor tissue and surrounding stroma tissue were naturally segmented in separate colors (tumor tissue in red, stroma tissue in green, “others” in white, shown in Figure 3). In the discovery cohort (*n* = 234), stroma‐low and stroma‐high were identified in 43.6% (*n* = 102) and 56.4% (*n* = 132) cases, respectively. The Kaplan–Meier curve showed that stroma‐high patients were associated with worse 5‐DFS compared to stroma‐low patients (*χ* ^2^ = 7.396, *p* = 0.007), which was consistent with previous studies.^26^ Also, our results proved that the pathologist annotated and algorithm predicted TSR had an outstanding consistency (ICC = 0.960, 95% CI 0.945–0.971), which indicated a favorable TSR assessment ability of the algorithm.

Having assessed TSR prognostic validity, we present a nomogram to enable an individualized prognostic prediction. Multivariable analysis identified age (*p* = 0.077), T stage (*p* = 0.030), N status (*p* < 0.001), histological grade (*p* < 0.001), ER status (*p* = 0.066), HER‐2 gene (*p* = 0.024) and TSR (*p* = 0.049) as potential risk predictors of invasive BC, which were included into the nomogram. The nomogram was well‐calibrated in the calibration plot and showed favorable predictive value (AUC 0.873 and 0.759) in the discovery and validation cohorts, respectively. In previous studies,^25^ the risk stratification ability of the prediction model is important because it's the premise of formulating treatment strategies. As a result, the sum points of each factor in the nomogram were calculated and Kaplan–Meier curves were used to estimate the probability of DFS between risk subgroups of two prediction models. As expected, a better risk stratification was found in the nomogram (*χ* ^2^ = 162.5, *p* < 0.001 for both I vs. II and II vs. III) than the TNM staging system (*χ* ^2^ = 57.0, *p* = 0.014 for Stage I vs. Stage II, *p* < 0.001 for Stage II vs. Stage III). In the external validation of the nomogram, this conclusion was further validated in Figure 8C and D.

The strengthening of our work is that it alleviates the workflow of investigators to manually categorize the histopathology to achieve an objective and fast TSR assessment. In addition, the algorithm can be applied to TSR assessment in regular microscopic fields, which is shown in Figure 2. The *TMA derrayer* module in the Qupath software also allows a high throughput TSR assessment in the TMAs. This indicates that with automated approaches, conventional histopathology images could be better used to facilitate the extraction of prognostic information to enable clinical prediction.^34^

While the QuPath algorithm used in our study performed reasonably well, it could only achieve rough segmentation without fully‐obtained tissue structure details, which was one of the main limitations of this study. For H&E‐stained images with different staining quality, the trained algorithm also required slight modification to achieve an optimal segmentation of tumor tissue and stroma tissue. Furthermore, although the TMAs were strictly constructed according to the criteria that only the most invasive tumor areas containing both tumor cells and stroma cells were selected, not every core of the TMAs could completely represent the optimal site for TSR assessment, which might cause selection bias. For the nomogram predicting survival of invasive BC patients, the sample size of the validated cohort was not large enough and the results might require a larger sample size for further validation.

## 5 CONCLUSIONS

In general, this study employed an open‐source software platform to develop an automatic TSR assessment algorithm based on the recognition of tumor and stroma tissues. The digital image analysis algorithm could provide a solution to the problems of observer variation caused by subjectivity, and the high throughput analysis alleviated the workflow of investigators. Furthermore, the established nomogram containing TSR provided a comprehensive individualized risk prediction strategy and might assist clinicians to make optimal care decisions for BC patients.

## AUTHORS' CONTRIBUTIONS

Conception and design: QX, BX and YHL. Data collection: JSZ and ZHL. Data analysis and interpretation: QX, YYC and LWW. Manuscript writing: QX and LWW. Final approval of manuscript: all authors. All authors read and approved the manuscript.

## FUNDING INFORMATION

This work was supported by the Young Scientists Fund of National Natural Science Foundation (Grant Numbers 81701768 and 81702901) and the Project for Young and Middle‐aged Medical Backbone Personnel of Wuhan City (Grant Number YL1400000032).

## CONFLICT OF INTEREST

The authors declare no conflict of interest.

## ETHICS APPROVAL

The study was approved by the Institutional Ethics Committee of Zhongnan Hospital of Wuhan University (Scientific Ethical Approval NO.2017057).

## CONSENT FOR PUBLICATION

Not applicable.

## Supporting information


Figure S1

Table S1
Table S2

## Data Availability

The datasets used and analyzed during the current study are available from the corresponding author (LWW) on reasonable request.
