# Supplementary figures and images for: Proposal of an automated tumor‐stromal ratio assessment algorithm and a nomogram for prognosis in early‐stage invasive breast cancer
Source: Cancer Med. 2022 Jun 11;12(1):131–45. doi: 10.1002/cam4.4928 (PMC9844605; doi:10.1002/cam4.4928)

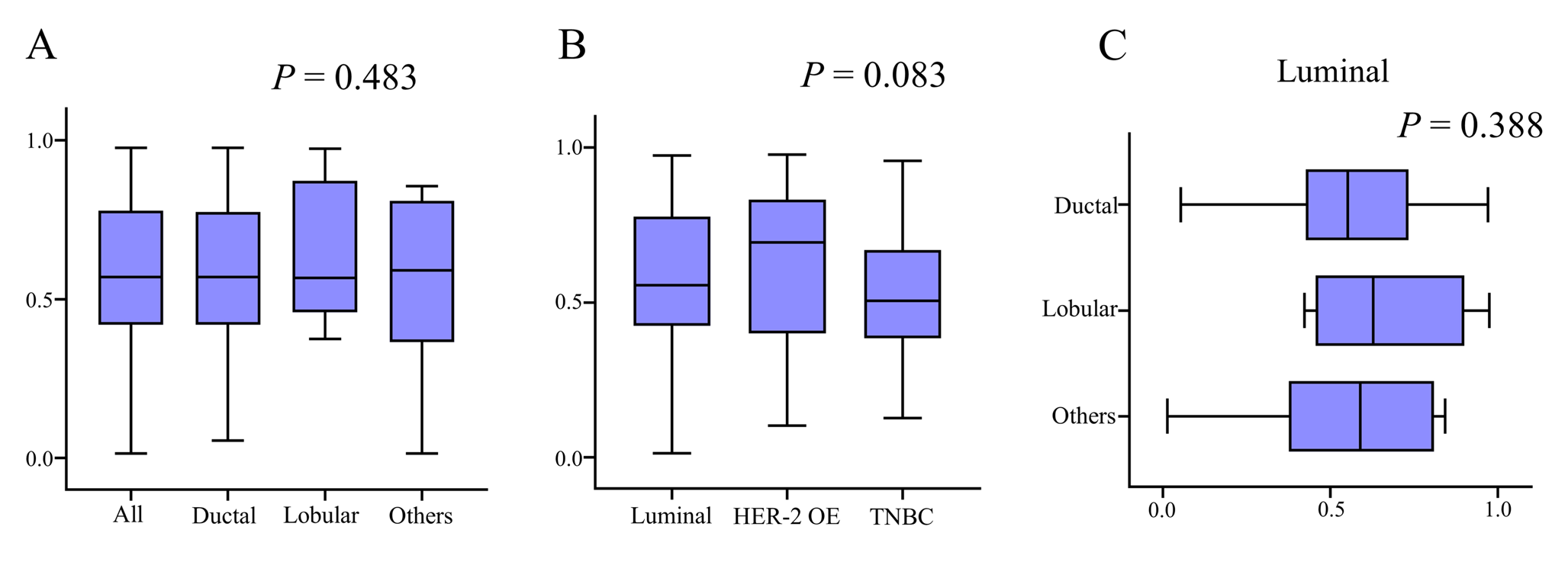

Supplement: Supplementary file 1 — Figure S1 [file CAM4-12-131-s001.tif]
