# Supplementary material for: Proposal of an automated tumor‐stromal ratio assessment algorithm and a nomogram for prognosis in early‐stage invasive breast cancer
Source: Cancer Med. 2022 Jun 11;12(1):131–45. doi: 10.1002/cam4.4928 (PMC9844605; doi:10.1002/cam4.4928)
Supplement: Supplementary file 2 — Table S1 Table S2 [file CAM4-12-131-s002.doc]

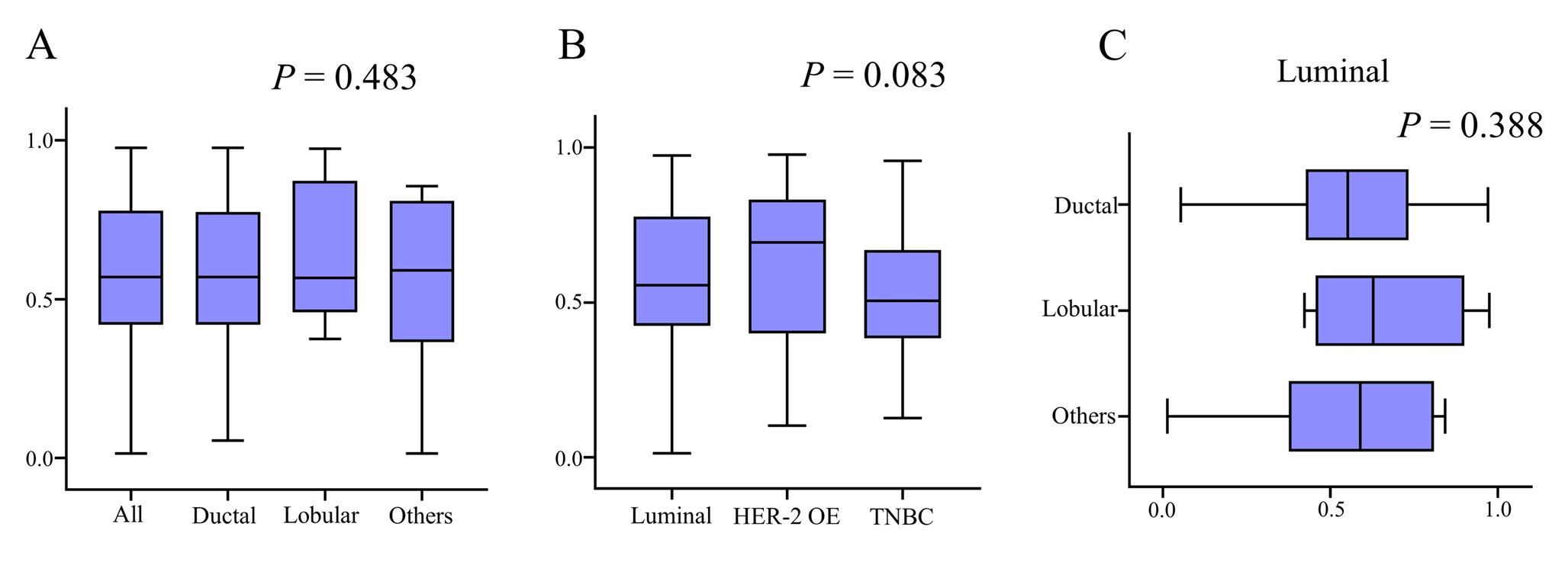


**Fig. S1** Correlation of the TSR with the molecular and histological types. HER2 OE: HER-2 overexpression.

| **Supplementary Table 1** List of features calculated for TSR assessment |
| --- |
| ROI: 2.00μm per pixel: OD Sum: Mean,  ROI: 2.00μm per pixel: OD Sum: Haralick Angular second moment,  ROI: 2.00μm per pixel: OD Sum: Haralick Contrast,  ROI: 2.00μm per pixel: OD Sum: Haralick Correlation,  ROI: 2.00μm per pixel: OD Sum: Haralick Sum of squares,  ROI: 2.00μm per pixel: OD Sum: Haralick Inverse difference moment,  ROI: 2.00μm per pixel: OD Sum: Haralick Sum average,  ROI: 2.00μm per pixel: OD Sum: Haralick Sum variance,  ROI: 2.00μm per pixel: OD Sum: Haralick Sum entropy,  ROI: 2.00μm per pixel: OD Sum: Haralick Entropy,  ROI: 2.00μm per pixel: OD Sum: Haralick Difference variance,  ROI: 2.00μm per pixel: OD Sum: Haralick Difference entropy,  ROI: 2.00μm per pixel: OD Sum: Haralick Information measure of correlation 1,  ROI: 2.00μm per pixel: OD Sum: Haralick Information measure of correlation 2,  ROI: 2.00μm per pixel: Hue: Mean  Smoothed: 50μm: ROI: 2.00μm per pixel: OD Sum: Mean,  Smoothed: 50μm: ROI: 2.00μm per pixel: OD Sum: Haralick Angular second moment,  Smoothed: 50μm: ROI: 2.00μm per pixel: OD Sum: Haralick Contrast,  Smoothed: 50μm: ROI: 2.00μm per pixel: OD Sum: Haralick Correlation,  Smoothed: 50μm: ROI: 2.00μm per pixel: OD Sum: Haralick Sum of squares,  Smoothed: 50μm: ROI: 2.00μm per pixel: OD Sum: Haralick Inverse difference moment,  Smoothed: 50μm: ROI: 2.00μm per pixel: OD Sum: Haralick Sum average,  Smoothed: 50μm: ROI: 2.00μm per pixel: OD Sum: Haralick Sum variance,  Smoothed: 50μm: ROI: 2.00μm per pixel: OD Sum: Haralick Sum entropy,  Smoothed: 50μm: ROI: 2.00μm per pixel: OD Sum: Haralick Entropy,  Smoothed: 50μm: ROI: 2.00μm per pixel: OD Sum: Haralick Difference variance,  Smoothed: 50μm: ROI: 2.00μm per pixel: OD Sum: Haralick Difference entropy,  Smoothed: 50μm: ROI: 2.00μm per pixel: OD Sum: Haralick Information measure of correlation 1,  Smoothed: 50μm: ROI: 2.00μm per pixel: OD Sum: Haralick Information measure of correlation 2,  Smoothed: 50μm: ROI: 2.00μm per pixel: Hue: Mean,  Smoothed: 50μm: Nearby detection counts |
|  |

| **Supplementary Table 2** The relationship between the TSR and clinicopathological factors in the validation cohort | | | | |
| --- | --- | --- | --- | --- |
| Characteristics | Total, n (%) | Stroma low, n (%) | Stroma high, n (%) | *P* value |
| **Age (years)** |  |  |  | 0.831 |
| ≤ 50 | 37 (36.6) | 20 (35.7) | 17 (37.7) |  |
| > 50 | 64 (63.4) | 36 (64.3) | 28 (62.3) |  |
| **Histological type** |  |  |  | 0.158 |
| No special type | 92 (91.1) | 49 (87.5) | 43 (95.5) |  |
| Others | 9 (8.9) | 7 (12.5) | 2 (4.5) |  |
| **T stage (cm)** |  |  |  | 0.244 |
| T1 (T ≤ 2) | 43 (42.6) | 21 (37.5) | 22 (48.9) |  |
| T2 (2 < T ≤ 5) | 57 (56.4) | 35 (62.5) | 22 (48.9) |  |
| T3 (T > 5) | 1 (1.0) | 0 (0) | 1 (2.2) |  |
| **N status** |  |  |  | 0.070 |
| N negative | 55 (54.5) | 35 (62.5) | 20 (44.4) |  |
| N positive | 46 (45.5) | 21 (37.5) | 25 (55.6) |  |
| **Histological grade** |  |  |  | 0.291 |
| I | 0 (0) | 0 (0) | 0 (0) |  |
| II | 83 (82.2) | 44 (78.6) | 39 (86.7) |  |
| III | 18 (17.8) | 12 (21.4) | 6 (13.3) |  |
| **ER status** |  |  |  | 0.199 |
| Negative | 31 (30.7) | 14 (25.0) | 17 (37.7) |  |
| Positive | 66 (65.3) | 39 (69.6) | 27 (60.0) |  |
| NA | 4 (4.0) | 3 (5.4) | 1 (2.3) |  |
| **PR status** |  |  |  | 0.823 |
| Negative | 48 (47.5) | 27 (48.2) | 21 (46.7) |  |
| Positive | 50 (49.5) | 27 (48.2) | 23 (51.1) |  |
| NA | 3 (3.0) | 2 (3.6) | 1 (2.2) |  |
| **HER2 gene** |  |  |  | 0.752 |
| Non-amplification | 77 (76.2) | 44 (78.6) | 33 (73.3) |  |
| Amplification | 17 (16.8) | 9 (16.1) | 8 (17.8) |  |
| NA | 7 (7.0) | 3 (5.3) | 4 (8.9) |  |
| BC: breast cancer; T: tumor; N: node; TSR: tumor-stromal ratio; ER: estrogen receptor; HER2: human epidermal growth factor receptor-2 | | | | |
